# Supplementary material for: Identification of Hub Genes and Key Pathways Associated with Sepsis Progression Using Weighted Gene Co-Expression Network Analysis and Machine Learning
Source: Int J Mol Sci. 2025 May 7;26(9):4433. doi: 10.3390/ijms26094433 (PMC12072417; doi:10.3390/ijms26094433)
Supplement: Supplementary file 1 [file ijms-26-04433-s001.zip › ijms-3540186-supplementary.pdf]

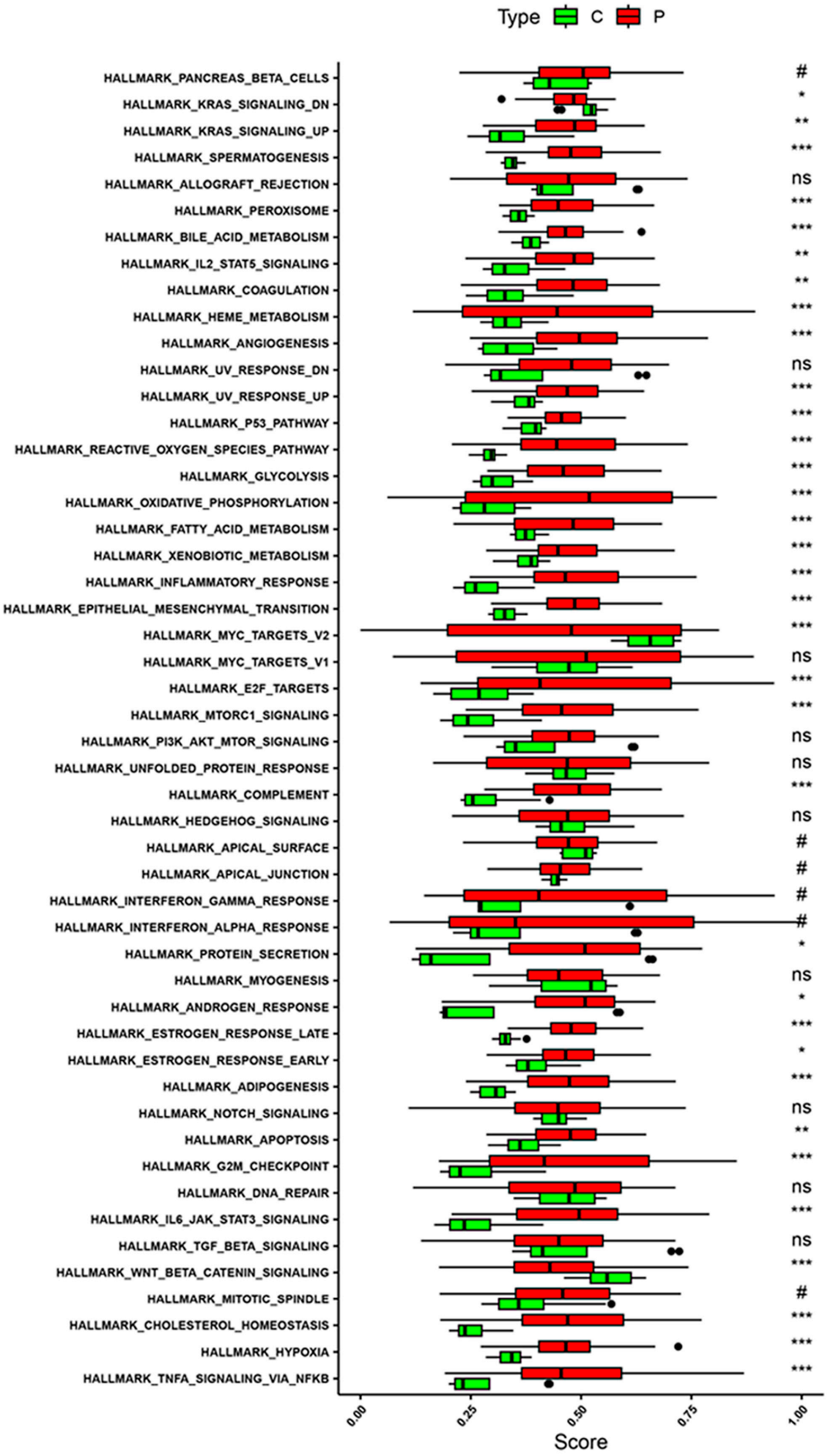

Figure S1. The heatmap shows normalized enrichment scores (NES) for representative pathways across sepsis and control samples.
